# An optimized cultivation method for future *in vivo* application of γδ T cells

**DOI:** 10.3389/fimmu.2023.1185564

**Published:** 2023-07-19

**Authors:** Anna Bold, Heike Gross, Elisabeth Holzmann, Stefan Knop, Timm Hoeres, Martin Wilhelm

**Affiliations:** ^1^ Department of Hematology and Medical Oncology, Paracelsus Medical University, Nuremberg, Germany; ^2^ Fraunhofer-Institute for Translational Medicine & Pharmacology (ITMP), Clinical Research, Frankfurt, Germany

**Keywords:** γδ T cells, Vγ9Vδ2 T cells, cellular immunotherapy, zoledronate, interleukin 2, interleukin 15, ADCC, cell culture

## Abstract

γδ T cells, with their properties of both the innate and acquired immune systems, are suitable candidates for cellular immunotherapy in cancer. Because of their non-major histocompatibility complex (MHC) binding T cell receptor, allogenic transfer is feasible without relevant graft versus host reactions. In recent years, much experience has been gained with *ex vivo* expansion and stimulation of γδ T cells using bisphosphonates and Interleukin 2. Unfortunately, many current stimulation protocols are based on the use of xenogenic materials and other potentially hazardous supplements, which conflicts with basic principles of Good Manufacturing Practice (GMP). Adherence to the concept and current guidelines of GMP is state of the art for production of Advanced Therapy Medicinal Products (ATMP) like cell therapeutics and a necessity for clinical use under a regulatory perspective. In this study, we developed a new stimulation protocol that induces a marked increase of γδ T cell counts and allows for an easier transition from research to clinical applications with minimized regulatory workload. It reliably leads to a cell product with a purity of more than 90% γδ T cells and improved *in vitro* anti-tumor activity compared to our previous standard procedure. Furthermore, by investigating correlations between properties of unstimulated γδ T cells and proliferation rate as well as degranulation ability of stimulated γδ T cells, we can draw conclusions about suitable donors. Finally, we examined if expansion can be improved by pulsing zoledronate and/or using Interleukin 15 with or without Interleukin 2. Significant improvements can be achieved with respect to intrinsic and antibody-dependent cell-mediated cytotoxicity. Our results demonstrate that the stimulation protocol presented here leads to an improved γδ T cell product for future clinical applications.

## Introduction

Cellular immunotherapy is becoming increasingly important in the treatment of cancer. One of the novel approaches for immunotherapy are γδ T cells. In contrast to αβ T cells, their T cell receptor (TCR) is not restricted to bind antigens in the context of a major histocompatibility complex (MHC) molecule. Part of the TCR are the variable Vγ and Vδ chains, with the Vγ9Vδ2 T cells being the dominant subpopulation and accounting for approximately 5% of T cells in peripheral blood (1). The anti-tumor effects of γδ T cells mediated by different mechanisms including cytokine production, perforin and interferon γ (IFNγ) release and antibody-dependent cell-mediated cytotoxicity (ADCC) have been demonstrated in detail *in vitro* and *in vivo* (2–5). Because of the MHC-independent recognition of target cells, no graft versus host reaction is to be expected with allogenic transfer of γδ T cells as has already been shown in several clinical studies (6–8).

Strategies to use γδ T cells as cellular therapy include activation of either the patient’s own γδ T cells or transferred allogenic γδ T cells *in vivo* or expansion and stimulation of autologous as well as allogenic γδ T cells *ex vivo* with subsequent adoptive transfer (6, 7, 9). The expansion and stimulation of Vγ9Vδ2 T cells both *in vivo* and *ex vivo* is usually achieved via aminobisphosphonates or natural or synthetic phosphoantigens and addition of co-stimulators, mostly cytokines, like Interleukin 2 (IL-2) (10, 11). The aminobisphosphonate zoledronate (Zol) inhibits the farnesyl pyrophosphate synthase enzyme in the mevalonate pathway of antigen presenting cells like monocytes, which consequently leads to accumulation of isopentenyl pyrophosphate (IPP). As natural phosphoantigens, IPP and its metabolites bind to butyrophilin 3A (BTN3A) molecules, which interact with BTN2A1 and are recognized by the TCR of Vγ9Vδ2 T cells with subsequent proliferation (12–14). A disadvantage of *in vivo* activation is the toxic effect of IL-2 at higher doses and of zoledronate when used at short intervals (10). Additionally, the *in vivo* stimulability of γδ T cells decreases with repeated administrations of the activators (15). However, the need of repetitive treatment is supported by our own clinical data. Indeed, the *in vivo* stimulation of haploidentical γδ T cells induced remission in most patients, but all of them relapsed within one year (6, 16). The number and functionality of the patient’s own γδ T cells is often limited (especially in cancer patients), so expansion and stimulation of these cells is usually unsuccessful both *in vivo* and *ex vivo* (10). For these reasons, we believe that *ex vivo* stimulation of healthy donor γδ T cells followed by adoptive transfer is the way forward.

In recent years, numerous cultivation methods have been published to stimulate γδ T cells *ex vivo*, but many of them use cell culture medium supplemented with fetal bovine serum (FBS). Using xenogeneic products bears the risk of contamination with known and even unknown pathogens and transmission of zoonotic diseases. In addition, such protocols often require culturing in plates and manual splitting, which is not compatible with a closed, sterile cultivation system advantageous for implementation of a GMP-compliant process. Thus, we developed a new stimulation protocol using a cell culture medium free of xenogenic products and serum and culturing in bottles without splitting. This can later be more easily expanded to the closed system of a cell growth device for clinical use without the need of fundamental adjustments.

The aim of this work was to evaluate and improve this newly developed protocol called “Ko-Op” with regard to the quantity and quality of the generated cell product. In addition to cell number and purity, we therefore investigated the cytoplasmic perforin and IFNγ in γδ T cells. Both proteins are produced in stimulated immune cells and have immunostimulatory and anti-tumor effects. We also determined CD107a on the surface of γδ T cells as a marker for degranulation and thus activation either as a result of sole stimulation or additionally in response to incubation with tumor cells. Furthermore, we measured the cytotoxicity of the stimulated γδ T cells against lymphoma cell line Daudi with or without monoclonal antibodies which induce ADCC (17, 18).

Despite a highly standardized cultivation procedure, stimulated γδ T cells from various donors differ in yield and anti-tumor activity when expanded *in vitro*. Therefore, we investigated if certain parameters prior to stimulation can indicate whether the cells are highly stimulable and activatable.

Finally, we examined if the cultivation procedure Ko-Op could be further improved. Nada et al. showed that improved proliferation rate and anti-tumor activity can be achieved by pulsed, high-dose zoledronate administration (19). Furthermore, some studies suggest that the addition of Interleukin 15 (IL-15) also leads to an improved proliferation rate and anti-tumor cytotoxicity of γδ T cells (20, 21). We therefore modified the cultivation protocol in several steps by using a zoledronate pulse instead of zoledronate standard and adding IL-15 instead of or additional to IL-2 and compared it with the standard procedure.

## Materials and methods

### Cell culture, cell isolation and *ex vivo* stimulation of γδ T cells

Our investigations were performed with peripheral blood obtained from healthy adult donors. The studies involving human participants were reviewed and approved by the Institutional Review Board of the Paracelsus Medical University Nuremberg. Written informed consent to participate in this study was provided by the participants. All donors signed an agreement according to General Data Protection Regulation.

Peripheral blood mononuclear cells (MNC) were isolated by density gradient centrifugation with Biocoll (Biochrom, Darmstadt, Germany/Bio&SELL, Feucht, Germany) and divided in several fractions in order to expand them using different cultivation methods.

To expand and stimulate γδ T cells we used our previously established protocol “R10F” and a new developed “Ko-Op”. MNC were incubated up to 17 days at 37°C and 5% CO_2_ in both protocols.

#### R10F

For cultivation according to the R10F protocol MNC at a concentration of 5.0E+05/ml were cultured in 96 U-bottom plates (Greiner bio-one, Frickenhausen, Germany) using standard medium consisting of RPMI 1640 supplemented with 10% fetal bovine serum (FCS), 1% 200mM L-glutamine and 1% penicilline/streptomycin (all from Biochrom, Darmstadt, Germany/Bio&SELL, Feucht, Germany). On day 0, 1 µM Zoledronate (Zol) (Sigma-Aldrich, St. Louis, USA) and 100 U/ml Interleukin 2 (IL-2) (Burton-on-Trent, Great Britain, United Kingdom) were added. From day 7, the cells were harvested twice a week, washed and reseeded at a concentration of 6.0E+05/ml in 96 well plates with fresh medium and 100 U/ml IL-2.

#### Ko-Op

For cultivation according to the Ko-Op protocol MNC at a concentration of 5.0E+05/ml were cultured in 50 ml cell culture flasks (Sarstedt, Nuembrecht, Germany) using medium consisting of OpTmizer™ CTS™ T-Cell Expansion Basal Medium, OpTmizer™ CTS™ T-Cell Expansion Supplement (Gibco/Thermo Fisher, Waltham, USA) and 1% 200 mM L-Glutamin. On day 0, 10 µM Zol were added and 1000 U/ml IL-2 were added on day 2. On day 4, 7, 9, 11 and 14 half of the medium was removed and replaced by fresh medium containing 2x 1000 U/ml IL-2 for a final concentration of 1000 U/ml. In some experiments we added 10 ng/ml Interleukin 15 (IL-15) (Peprotech, Cranbury, USA) instead of or in addition to 1000 U/ml IL-2. Cells were not split at any time. From day 4 on, cell culture flasks were shaken at 250 rpm. For pulsing zoledronate, 100 µM Zol were added on day 0. After 4 hours incubating at 37°C and 5% CO_2_ MNC were harvested, washed twice and reseeded at a concentration of 5.0E+05/ml in cell culture flasks with fresh medium. The addition of IL-2 and/or IL-15 from day 2 and further cultivation was done as described above.

The lymphoma cell line Daudi was obtained from the German collection of microorganisms and cell culture (DSMZ, Braunschweig, Germany) and cultured in our standard medium R10F.

Cell counts and cell viability were established using a hemocytometer and the trypan blue exclusion method. Cell count and proliferation rate of γδ T cells was calculated on the basis of the cell number of the MNC and the percentage of γδ T cells on the MNC determined by flow cytometry.

### Flow cytometry and antibodies

A FC500 and a Navios flow cytometer (both Beckman Coulter, Brea, USA) were used for multicolor immunofluorescence and functional tests.

Cells were stained in appropriate combinations according to use with following antibodies: anti-T-cell receptor (TCR) γδ-FITC [clone IMMU510], anti-CD3-r-Phycoerythrin-Texas Red (ECD) [clone UCHT1], anti-CD56-phycoerythrin-cyanine 5 (PC5) [clone N901], anti-CD27-phycoerythrin-cyanine 5 (PC5) [clone 1A4CD27], anti-CD56-phycoerythrin-cyanine 7 (PC7) [clone N901] (all Beckman Coulter, Brea, USA); anti-T-cell receptor (TCR) γδ-FITC [clone 11F2], anti-CD107a-PE [clone H4A3], anti-IFNγ−PE [clone 45-15], anti-CD45RA-PE [clone REA 562] (all Miltenyi Biotec, Bergisch Gladbach, Germany); and anti-perforin-PE [clone B-D48] (Biolegend, San Diego, USA).

As negative control, anti-IgG-PE [clone IS5-32F5] (Miltenyi Biotec, Bergisch Gladbach, Germany) was used.

γδ cells were defined as CD3^+^ TCR γδ^+^ MNC, NK cells as CD3^-^ CD56^+^ MNC and αβ T cells as CD3^+^ TCR γδ^-^ MNC.

For functional assay, the therapy grade monoclonal antibodies rituximab and obinutuzumab (both Roche, Grenzach-Wyhlen, Germany) as well as the IgG1-kappa control antibody (Sigma-Aldrich, St. Louis, USA) were used.

### Cytoplasmic staining

After staining the surface antigens with anti-TCR γδ-FITC and anti-CD3-ECD, intracellular IFNγ or intracellular perforin were stained with anti-IFNγ-PE or anti-perforin-PE or anti-IgG-PE as control using the inside stain kit (Miltenyi Biotec, Bergisch Gladbach, Germany) according to manufacturer’s instructions. Afterwards, the cells were analyzed by flow cytometry. To define perforin^+^ or IFNγ^+^ γδ T cells, cells were stained with an isotype control and the gate was adjusted so that 2% of the cells in the isotype control were defined as positive. ΔMFI was calculated as MFI (IFNγ or perforin) minus MFI (isotype control).

### Degranulation assay

The surface antigen CD107a as marker for degranulation was determined in our degranulation assay. For this, stimulated MNC were co-cultured with Daudi in a 1:2 ratio or with the respective medium with the addition of the anti-CD107a-PE or anti-IgG-PE as control in 96 well V-bottom plates (Greiner bio-one, Frickenhausen, Germany) for 3h. Subsequently, the cells were washed, the surface antigens were stained with anti-TCR γδ-FITC and anti-CD3-ECD and the cells were analyzed by flow cytometry. The definition of CD107a^+^ γδ T cells as well as the determination of the ΔMFI of CD107a was performed analogously to the procedure for perforin and IFNγ.

### Immuno-magnetic depletion

MNC stimulated with R10F and Ko-Op consist mainly of γδ T cells, but especially after stimulation with R10F also of αβ T cells and NK cells. To compare the cytotoxicity of γδ T cells when cultured with R10F or Ko-Op, αβ T cells and NKp46^+^ cells were depleted from stimulated MNC after 10 days of cultivation using the MidiMACS system and anti-TCRαβ and anti-NKp46 MicroBeads (all from Miltenyi Biotec, Bergisch Gladbach, Germany) according to manufacturer’s instructions except using HSA (CSL Behring GmbH, Marburg, Germany) instead of BSA as part of the washing buffer. In order to proof the success of depletion, the cells were stained before and after depletion with anti-TCR γδ-FITC, anti-CD3-ECD and anti-CD56-PC5 to diversify the different cell populations. Cell counts and cell viability were determined using a hemocytometer and the trypan blue exclusion method.

### Cytotoxicity assay

Cytotoxicity experiments were conducted in 96 well V-bottom plates (Greiner bio-one, Frickenhausen, Germany) as co-cultures at different effector to target cell ratios with 1 µg/ml rituximab, 1 µg/ml obinutuzumab or 1 µg/ml isotype control antibody. Daudi were used as target cells and previously labeled with carboxyfluorescein succinimidyl ester (CFSE) (BioLegend, San Diego, USA) and taken up into the respective culture medium (R10F or OpTmizer™ CTS™ T-Cell Expansion Medium). Effector cells, which differed in regard to stimulation protocol, were used in this assay. If indicated, TCRαβ^+^ and NKp46^+^ cells were depleted as described above before performing the assay. Following co-culture of effector and target cells for 4 h, cells were harvested, technical replicates were pooled, treated with 7-AAD (Beckman Coulter, Brea, USA) and analyzed by flow cytometry. Specific cell mediated cytotoxicity is expressed as “specific lysis %” and calculated by the formula: specific lysis % = [% 7-AAD^+^ target cells in the respective effector to target ratio – % 7-AAD^+^ target cells in target cell only culture] *100/[100 – % 7-AAD^+^ target cells in target cell only culture]. The lytic units per 10^6^ effector cells were calculated according to Bryant et al. with the following formula: $lytic\;units\;per\;10^{6}\;effector\;cells=10^{6}\cdot exp\left(\frac{\bar{Y^{*}}-\;Y_{p}^{*}}{C}\right)/(T\cdot \bar{X_{G}}$ ) (22). *Y* is the specific lysis measured in a defined effector to target ratio. *Y* is logistically transformed (Y*) by following formula: $Y^{*}=LN\left(\frac{Y}{100-Y}\right)$ . We defined a reference lysis p of 60%, which is also logistically transformed $\left(Y_{p}^{*}\right)$ . $\;\bar{Y^{*}}$ is the arithmetic mean of the logistically transformed specific lyses measured in each effector to target ratio, C is a constant with relation to the slope of the curve (defined as 1), T is the number of target cells (10^4^), and $\bar{X_{G}}$ is the geometric mean of the effector to target ratios used in the assay.

### Data and statistical analysis

Data were analyzed with the software Kaluza analysis V2.1 (Beckman Coulter, Brea, USA), Excel 2016 (Microsoft, Redmond, USA) and SPSS Statistics 22 (IBM, Armonk, USA). Data are presented as mean ± standard deviation (SD). The normal distribution of the data was verified using the Shapiro test. Levels of significance were calculated using the paired t-test or the Wilcoxon test. Correlation coefficient R was calculated according to Spearman. p<0.05 is considered statistically significant.

## Results

### Proliferation, purity and anti-tumor efficiency of γδ T cells by using Ko-Op for *ex vivo* stimulation

To establish a GMP-compliant and effective cultivation of γδ T cells, we developed a new protocol called “Ko-Op”. For this, we replaced our previous cultivation media “R10F”, which contains FBS, with the xeno-free OpTmizer™ CTS™ T cell expansion medium. In order to achieve an optimal result in terms of yield, we further increased the concentrations of zoledronate and IL-2 and did not administer the first IL-2 addition until day 2 as it is already done successfully (23). Furthermore, we used flasks instead of 96 well plates to facilitate upscalability. Assuming that the nutrient supply to the cells is limited by diffusion due to sedimentation of cells in standing flasks, we placed them on an orbital shaker from day 4 of cultivation, but not earlier because of the need of the cell-cell contact between monocytes and γδ T cells for stimulation. **Table 1** shows the relevant differences to our established protocol R10F (17, 24).

**Table 1:** Relevant differences of the protocols R10F and Ko-Op for *ex vivo* stimulation of γδ T cells.

	R10F	Ko-Op
**Media**	RPMI 1640 Media	OpTmizer™ T-Cell Expansion SFM
**Supplements**	Fetal bovine serum, Glutamine, Penicillin/Streptomycin	Glutamine
**Culture plate**	96-well U-plate (200 µl/well)	50 ml flask (10 ml/flask)
**Stimulants**	1 µM Zol d0 100 U/ml IL-2 d0, d7, d10, d14	10 µM Zol d0 1000 U/ml IL-2 d2, d4, d7, d9, d11, d14
**Others**		shaker from d4

**Figure 1:**
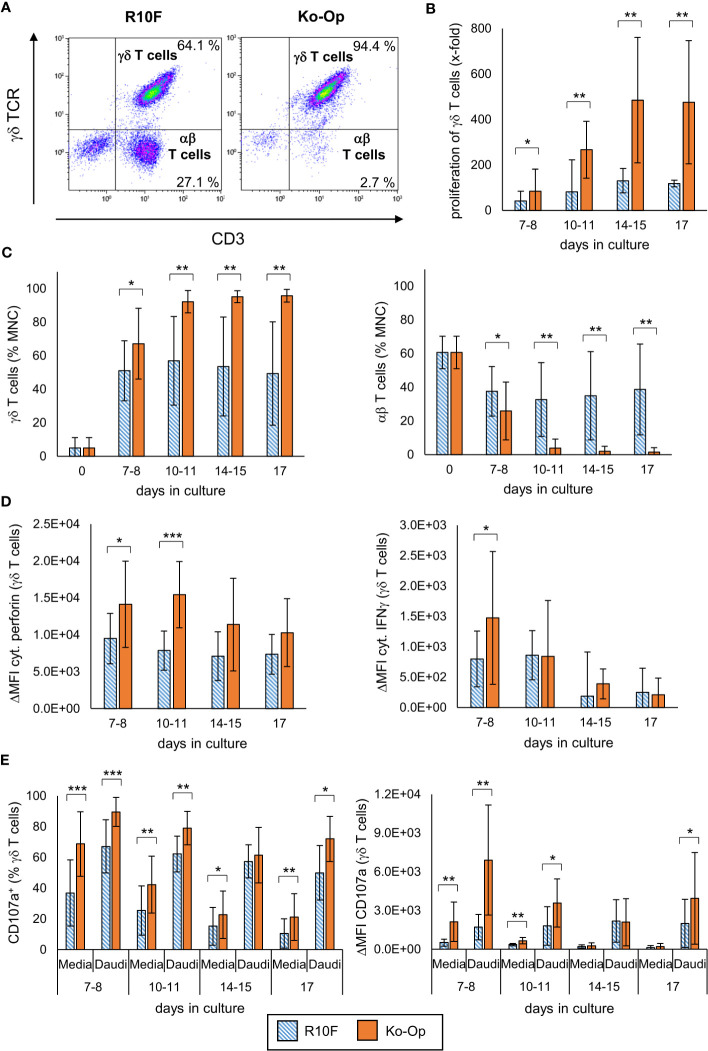
Proliferation, purity and anti-tumor efficiency of γδ T cells by using Ko-Op for *ex vivo* stimulation. MNC of healthy donors were isolated and stimulated with Zol/IL-2 according to the protocols R10F (blue hatched bars) and Ko-Op (orange bars) up to 17 days. **(A)** Representative FACS analysis of MNC cultured according to the protocols R10F or Ko-Op for ten days. γδ T cells and αβ T cells were defined by using anti-γδ TCR-FITC and anti-CD3-ECD. **(B)** Proliferation rate of γδ T cells calculated on the basis of the cell number of the MNC and the percentage of γδ T cells on the MNC determined by flow cytometry. **(C)** Percentage of γδ T cells and αβ T cells at different days of cultivation measured by flow cytometry. **(D)** Cytoplasmic perforin and IFNγ in γδ T cells were stained at different days of cultivation and measured by flow cytometry. ΔMFI is calculated as MFI (perforin or IFNγ) minus MFI (isotype control). **(E)** MNC were incubated with media control or Daudi at different days of cultivation in order to perform the degranulation assay. After 3h CD107a^+^ γδ T cells and ΔMFI of CD107a on γδ T cells were detected by flow cytometry. The data are presented as mean ± SD of 12 **(B, C)** or 9 **(D, E)** independent experiments. *p<0.05, **p<0.01 and ***p<0.001 comparing the two different stimulation protocols.

MNC of twelve healthy donors were isolated and stimulated with Zol/IL-2 using the protocols R10F and Ko-Op over 17 days. Every three to four days, the number of MNC was counted and the percentage of γδ T cells, αβ T cells and NK cells was determined by flow cytometry as exemplary shown in **Figures 1A**, **S1A**. In terms of γδ T cells, an average proliferation rate of more than 400-fold of the baseline can be achieved (**Figure 1B**). The percentage of γδ T cells is significantly higher when Ko-Op was used for cultivation (**Figure 1C**). Very strikingly, there was clearly less inter-donor variability when using Ko-Op compared to R10F. Additionally, the percentage of αβ T cells and NK cells is significantly lower when stimulated with Ko-Op also with low inter-donor variability (**Figures 1C**, **S1B**). The question arises if the higher doses of zoledronate and IL-2 within the Ko-Op protocol leads to reduced viability. Therefore, the vitality of MNC was determined by trypan blue exclusion test showing no statistical difference between the two different cultivation protocols (**Figure S1C**). A detailed viability analysis using annexin/7AAD staining performed on a part of the experiments also did not reveal an increased rate of apoptosis or dead cells when stimulated with Ko-Op compared to R10F (data not shown).

To evaluate the activity of stimulated γδ T cells, cytoplasmic perforin and IFNγ were stained every three to four days and the percentage of perforin^+^ and IFNγ^+^ γδ T cells as well as the ΔMFI was determined by flow cytometry. γδ T cells produced significantly more perforin when cultured according to the Ko-Op protocol (**Figures 1D**, **S1D**). With regard to IFNγ, the ΔMFI was only significantly increased on day 7 and the percentage of IFNγ^+^ γδ T cells did not differ between the two cultivation protocols (**Figures 1D**, **S1D**).

**Figure 2:**
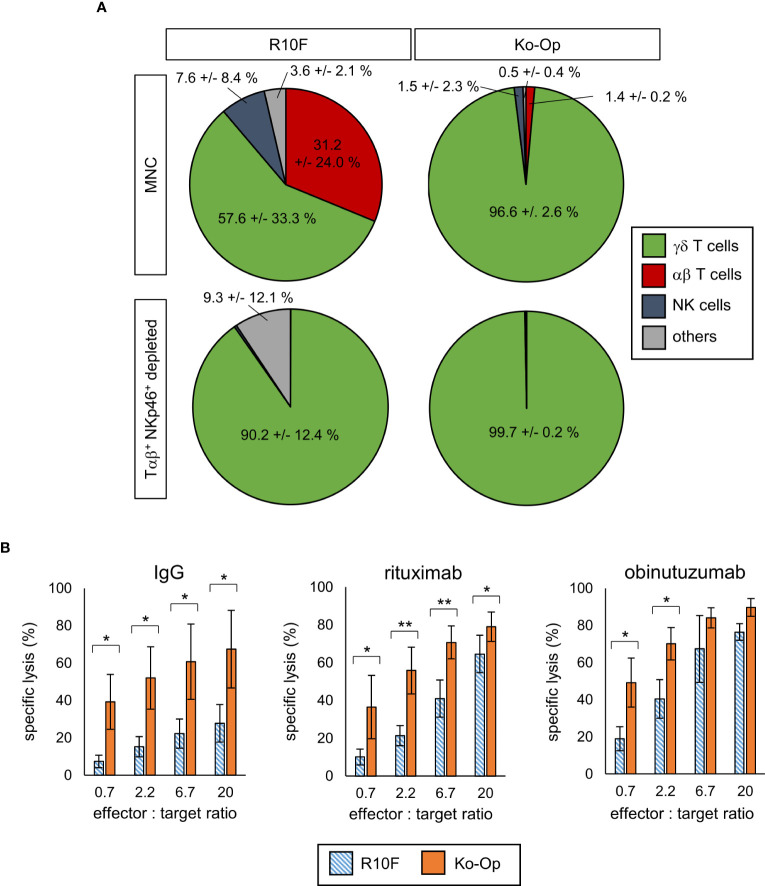
Cytotoxicity of γδ T cells by using Ko-Op for *ex vivo* stimulation. MNC of healthy donors were isolated and stimulated with Zol/IL-2 according to the protocols R10F and Ko-Op. On day 10 of cultivation, the paramagnetic depletion of Tαβ^+^ and NKp46^+^ cells was performed. **(A)** Composition of undepleted and Tαβ^+^ and NKp46^+^ depleted MNC on day 10. **(B)** Depleted MNC were incubated with Daudi and the monoclonal antibodies rituximab, obinutuzumab or their unspecific isotype control (IgG) for 4h in different effector to target ratios. Specific target cell lysis was measured by flow cytometry. The data are presented as mean ± SD of 4 **(A)** or 3-4 **(B)** independent experiments. *p<0.05 and **p<0.01 comparing the two different stimulation protocols.

For investigation of the anti-tumor efficiency of stimulated γδ T cells, degranulation of γδ T cells after 3h incubation with the tumor cell line Daudi or media only was determined by measuring the surface molecule CD107a by flow cytometry from day 7 onwards every three to four days. With and without tumor cells, stimulation with Ko-Op increased both the percentage of CD107a^+^ γδ T cells and the amount of CD107a on the surface of γδ T cells as determined by ΔMFI compared to stimulation with R10F (**Figure 1E**). To directly determine the anti-tumor activity of γδ T cells against the tumor cell line Daudi, we performed cytotoxicity assays on day 10 of cultivation. For this, αβ T cells and NKp46^+^ cells were paramagnetically depleted from the cell suspension in order to isolate the γδ T cells on day 10. The cell composition before and after depletion is shown **Figure 2A**. By cultivation using the Ko-Op protocol compared to R10F, a significant increase in the cytotoxicity of γδ T cells against Daudi could be achieved with both the monoclonal antibodies rituximab and obinutuzumab and their non-specific isotype control (**Figure 2B**). The specific lysis of Daudi cells under addition of IgG corresponds to the intrinsic cytotoxicity of γδ T cells as the unspecific isotype control does not mediate ADCC. To confirm this, we tested if there is a difference in specific lysis of Daudi between addition of IgG or no isotype control and could not find a significant difference both when stimulated with R10F or Ko-Op (data not shown). In summary, cultivation with Ko-Op enhances the proliferation rate, the purity, the anti-tumor activity and the cytotoxicity of stimulated γδ T cells.

**Figure 3:**
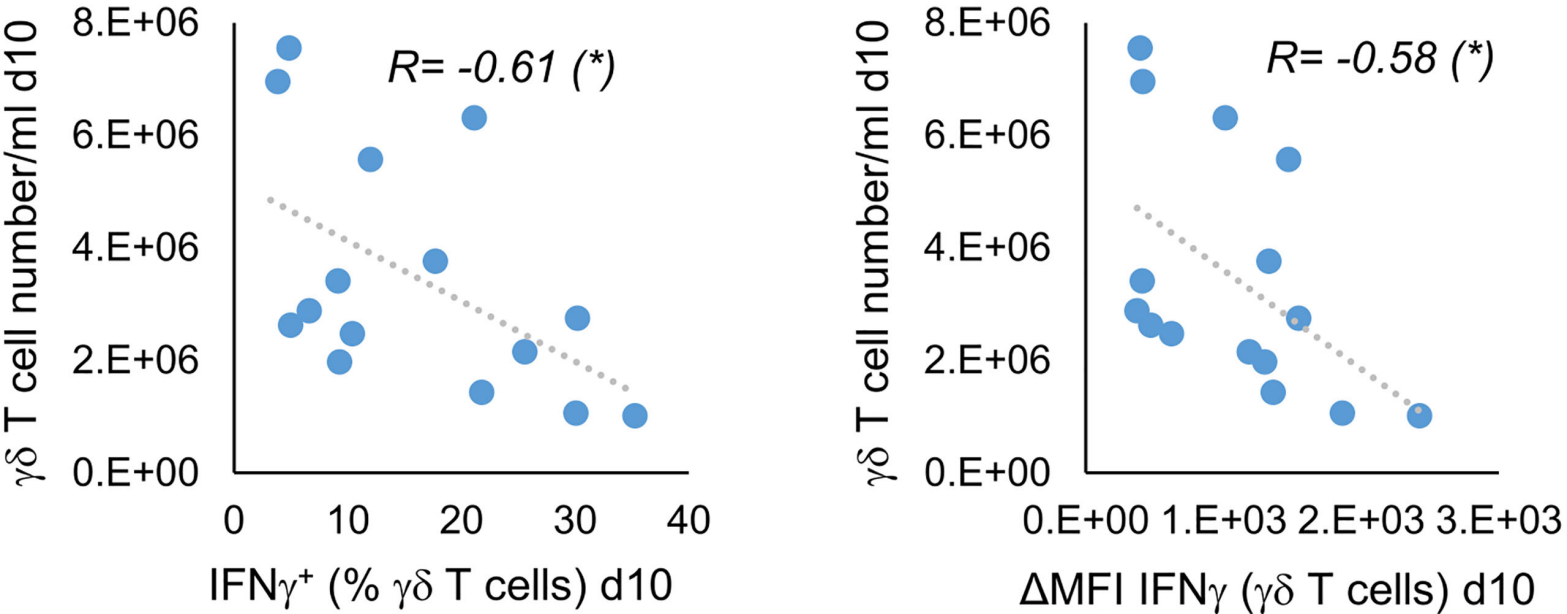
Indirect correlation of cell concentration and IFNγ production of stimulated γδ T cells. MNC of healthy donors were isolated and stimulated with Zol/IL-2 according to the protocol Ko-Op. On day 10, the γδ T cell number/ml was calculated on the basis of cell number/ml of MNC and percentage of γδ T cells on the MNC. The cytoplasmic IFNγ in γδ T cells was determined by flow cytometry. The results were tested for correlation. The data are presented as correlation chart of 15 independent experiments. Correlation coefficient R is calculated according to Spearman. *p<0.05 correlating the indicated variables.

Since we did not see a clear improvement in IFNγ production by stimulation with Ko-Op compared with R10F, we wondered whether all tested parameters could be equally optimized by stimulation. We therefore tested the proliferation rate of MNC, the percentage of γδ T cells, the CD107a expression and the cytoplasmic IFNγ on day 10 for correlations. While, as expected, the proliferation rate and purity as well as IFNγ production and CD107a expression were significantly related (data not shown), there was a negative correlation between cell concentration of γδ T cells and IFNγ production (**Figure 3**). Since we did not split the cell cultures during the cultivation with Ko-Op, the cell concentration is a direct indicator of cell count of γδ T cells. Thus, not all target parameters can be equally enhanced with our cultivation method.

### Correlation of donor characteristics with the proliferation and anti-tumor activity of their stimulated γδ T cells

**Figure 4:**
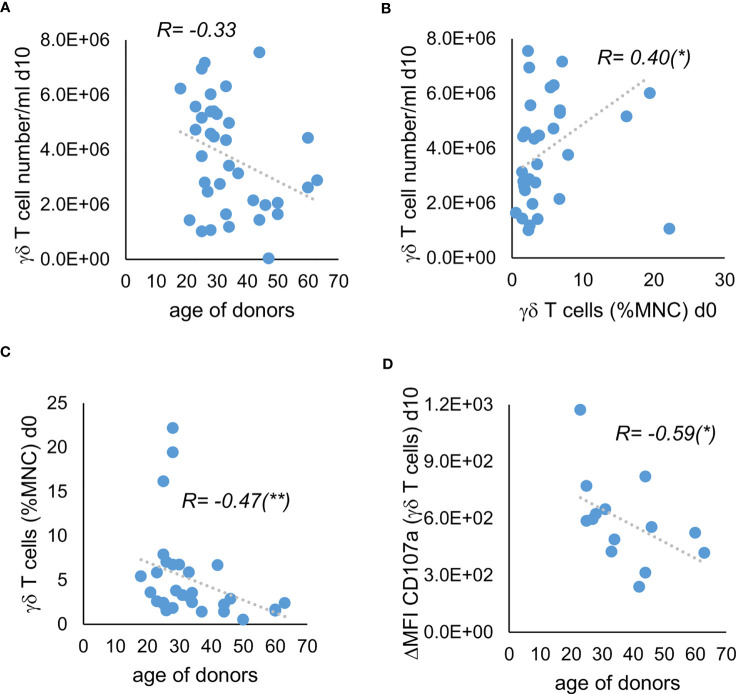
Correlation of donor characteristics with the proliferation and anti-tumor activity of their stimulated γδ T cells. MNC of healthy donors were isolated and stimulated with Zol/IL-2 according to the protocol Ko-Op. The percentage of γδ T cells was determined by flow cytometry. On day 10 of cultivation, the degranulation assay was performed. **(A)** Correlation between the age of donors and the γδ T cell number/ml on day 10 of stimulation. **(B)** Correlation between the percentage of γδ T cells when unstimulated and the γδ T cell number/ml on day 10 of stimulation. **(C)** Correlation between the age of the healthy donors and the percentage of γδ T cells when unstimulated. **(D)** Correlation between the age of the healthy donors and the ΔMFI of CD107a on γδ T cells on day 10 of stimulation. The data are presented as correlation chart of 36 **(A–C)** or 14 **(D)** independent experiments. Correlation coefficient R is calculated according to Spearman. *p<0.05 and **p<0.01 correlating the indicated variables.

Especially when multiple donors are available, it would be helpful to be able to deduce from the demographic or clinical characteristics of the donors or their unstimulated γδ T cells whether their stimulated γδ T cells have the potential for high yield and high anti-tumor activity. We could not find a significant correlation between the donors’ sex and the cell concentration of γδ T cells on day 10 of stimulation (data not shown). There was also no significant correlation between cell concentration at day 10 and the donors’ age, although the significance level for the negative correlation between age and cell concentration of γδ T cells was just not reached with a p value of 0.052 (**Figure 4A**). However, we observed a positive correlation between the cell concentration of γδ T cells on day 10 of stimulation and the percentage of γδ T cells of unstimulated MNC, which in turn correlated negatively with age (**Figures 4B, C**). Additionally, we found a negative correlation between age of donors and the ΔMFI of CD107a in the degranulation assay without incubation with Daudi cells (**Figure 4D**). In conclusion, higher age seems to be associated with lower percentage of γδ T cells in MNC and therefore with lower proliferation when stimulated by using Ko-Op. Additionally, the stimulated γδ T cells of older donors probably bear less anti-tumor activity compared to them of younger donors. Thus, the percentage of γδ T cells as well as the age of the donors can be used as a decision-making aid in the selection of donors.

### Modification of proliferation, purity and anti-tumor activity of γδ T cells by altering the stimulants within the Ko-Op protocol

We next investigated whether the Ko-Op protocol could be improved by a zoledronate pulse or by applying IL-15 in addition to or in place of IL-2, or by the combination of both. For this, MNC of healthy donors were isolated and stimulated using the Ko-Op standard protocol or according to different alterations concerning the zoledronate addition and the composition of the interleukins up to ten days. For zoledronate pulse, 100 µM zoledronate was added to the isolated MNC and washed out again after 4 hours of incubation. When IL-15 was used, this was at a concentration of 10 ng/ml, with the concentration of IL-2 remaining unchanged at 1000 U/ml.

**Figure 5:**
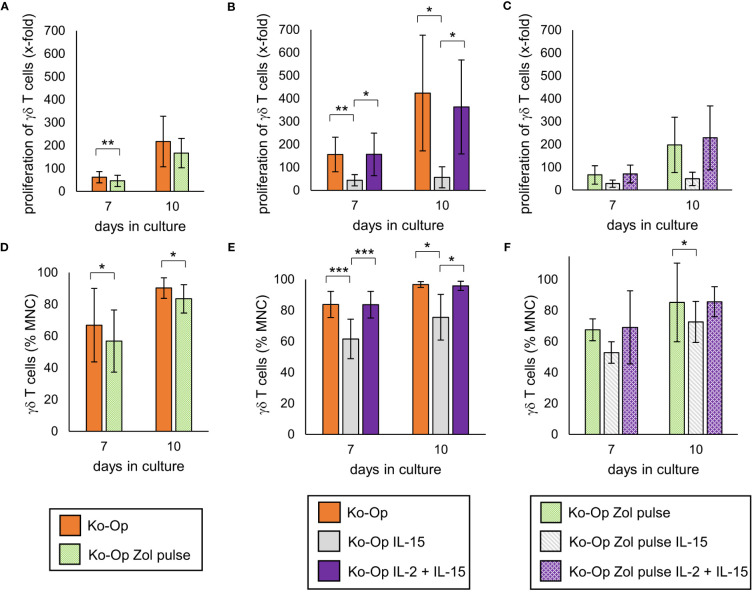
Modification of proliferation, purity and anti-tumor activity of γδ T cells by altering the stimulants within the Ko-Op protocol. MNC of healthy donors were isolated and stimulated according to the Ko-Op standard protocol (orange bars) or to different alterations concerning the zoledronate addition (Zol pulse) and the composition of the interleukins (IL-15, IL-2 + IL-15) up to ten days. **(A–C)** Proliferation rate of γδ T cells at day 7 and 10 of cultivation. **(D–F)** Percentage of γδ T cells at day 7 and 10 of cultivation measured by flow cytometry. The data are presented as mean ± SD of 7 **(A, D)**, 6 **(B, E)** or 4 **(C, F)** independent experiments. *p<0.05, **p<0.01 and ***p<0.001 comparing the different cultivation protocols.

Pulsing the cells with high-dose zoledronate led to a significant lower proliferation rate of γδ T cells on day 7 of cultivation (**Figure 5A**). While compared to the standard protocol, the combined application of IL-2 and IL-15 led to no significant change in proliferation rate of γδ T cells, the proliferation was significantly lower when IL-15 was used alone (**Figure 5B**). The combination of zoledronate pulse and adding IL-2 and IL-15 also did not lead to any improvement (**Figure 5C**). The proportion of γδ T cells and thus the purity of the cell product was significantly reduced by pulsing zoledronate, by using IL-15 instead of IL-2 and by combining both modifications (**Figures 5D–F**).

**Figure 6:**
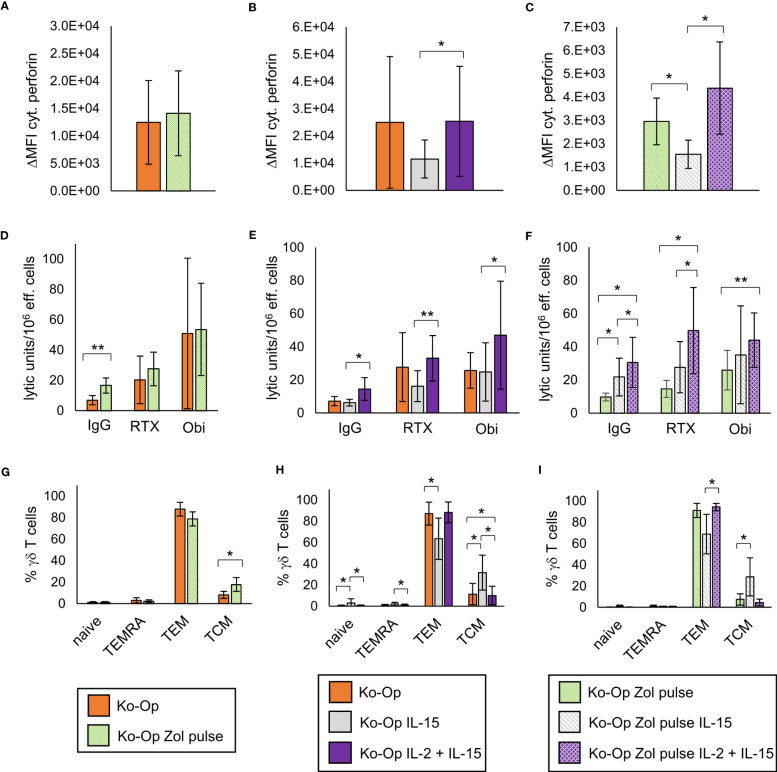
Modification of anti-tumor activity and differentiation of γδ T cells by altering the stimulants within the Ko-Op protocol. MNC of healthy donors were isolated and stimulated according to the Ko-Op standard protocol (orange bars) or to different alterations concerning the zoledronate additon (Zol pulse) and the composition of the interleukins (IL-15, IL-2 + IL-15) up to ten days. **(A–C)** Cytoplasmic perforin of γδ T cells was stained at day 10 of cultivation. ΔMFI was measured by flow cytometry. **(D–F)** MNC were incubated with Daudi with the monoclonal antibodies rituximab, obinutuzumab or their unspecific isotype control for 4h in order to perform the cytotoxicity assay at day 10 of cultivation. Lytic units per 10^6^ effector cells were calculated based on the specific target cells lysis obtained with different effector to target ratios from 0.7:1 to 20:1. **(G–I)** Percentage of the γδ T cells differentiated in naive (CD45RA^+^CD27^+^), TCM (CD45RA^-^CD27^+^), TEM (CD45RA^-^CD27^-^) and TEMRA (CD45RA^+^CD27^-^). The data are presented as mean ± SD of 5 **(A, F, G)**, 6 **(B, E, H)** or 4 **(C, D, I)** independent experiments. *p<0.05 and **p<0.01 comparing the different cultivation protocols.

Regarding cytoplasmic perforin, the use of IL-15 instead of IL-2 led to a significant reduction both without and with zoledronate pulse (**Figures 6B, C**). The zoledronate pulse alone and the use of IL-15 in addition to IL-2 did not lead to any significant change (**Figures 6A–C**). The modifications of the Ko-Op protocol had no significant effect on cytoplasmic IFNγ and CD107a expression in the degranulation assay (data not shown). We next performed cytotoxicity assays with the different cultivated MNC. To be able to compare the methods more accurately, we calculated the lytic units per 10^6^ effector cells according to Bryant et al. (22). A lytic unit is the number of effector cells, which is required to lyse a specific percentage of target cells. A significant increase in lytic units could be achieved with the zoledronate pulse compared to the standard protocol when no therapeutic antibodies are added (**Figure 6D**). The combination of IL-15 and IL-2 led to no change in lytic units both with and without antibodies compared to the standard protocol, but to significant higher lytic units compared to IL-15 alone (**Figure 6E**). Interestingly, when the modifications were combined, an increase in lytic units could be achieved without antibody when IL-15 alone or IL-15 and IL-2 were added, and with antibody when IL-15 and IL-2 were added, compared to the zoledronate pulse without modification of the interleukins (**Figure 6F**).

As IL-15 is known to promote the stimulation of central memory T cells, we analyzed certain subclasses of stimulated γδ T cells. γδ T cells were classified into naive, central memory, effector memory and terminally differentiated γδ T cells based on the expression of CD45RA and CD27 (20, 25). As expected, the stimulation with IL-15 instead of IL-2 led to an elevated percentage of central memory γδ T cells (TCM) and to a reduced percentage of effector γδ T cells (TEM), also if combined with pulsing zoledronate (**Figures 6H, I**). Pulsing zoledronate and addition of IL-2 as stimulant also resulted in more TCM (**Figure 6G**). Interestingly, combining IL-2 with IL-15 significantly reduced the percentage of TCM, but the difference was marginal (**Figure 6H**).

In conclusion, pulsing zoledronate and co-stimulation by the combination of IL-2 and IL-15 lead to significant changes in *in vitro* anti-tumor activity and in the subclasses of stimulated γδ T cells.

## Discussion

### Improvement of immunotherapeutic cell products by a newly developed cultivation method

The aim of this work was to lay the groundwork for a GMP-compliant cultivation process for γδ T cells that would reliably produce a cell product with high proliferation rate, purity and anti-tumor activity. By combining known and partly new process steps like shaking, we established the xenogen-free cultivation protocol “Ko-Op”. When stimulating MNC with this cultivation method, γδ T cell counts increased more than 400-fold on average and the cell product consisted of over 90% γδ T cells. Compared to our previous standard procedure, cultivation with Ko-Op led to higher production of cytoplasmic perforin, to higher degranulation and to stronger cytotoxicity with and without monoclonal antibodies directed against target cells. However, stimulation with Ko-Op increased cytoplasmic IFNγ only on day 7 of cultivation compared with our previous standard protocol “R10F”. IFNγ is a pro-inflammatory cytokine and its production is a typical hallmark of activated Vγ9Vδ2 T cells associated with their effector functions in cancer (26, 27). Interestingly, for γδ T cells stimulated with Ko-Op in our study, we observed a negative correlation between the proliferation rate at day 10 and both the proportion of cytoplasmic IFNγ expressing γδ T cells and the ΔMFI of cytoplasmic IFNγ in γδ T cells. Possibly, the IFNγ producing γδ T cells are distinctively differentiated and proliferate less upon stimulation by IL-2. Consistent with this, CD27^+^ γδ T cells were found to proliferate, but to have a reduced capacity to secrete IFNγ (28).

That OpTmizer is a suitable medium for serum-free expansion of γδ T cells has also been shown by Sutton et al. (29). Interestingly, the purity of γδ T cells after stimulation with Ko-Op in our study is notably higher than the described percentage of γδ T cells in their study. This could be due to the fact that we use a higher dose of zoledronate, do not split and shake the cells from day 4 onwards. However, we know that shaking is not the main reason for the higher purity as stimulation with Ko-Op without shaking leads to comparable purity but reduced vitality of MNC compared to Ko-Op standard stimulation (data not shown). We do not add IL-2 before d2. This could explain why the percentage of NK cells, which are known to proliferate upon IL-2 stimulation, is comparatively low after stimulation with Ko-Op. While the presence of NK cells might not negatively influence the anti-tumor activity of the final cell product and even be beneficial in some indications (30), the αβ T cells cause graft versus host reaction and must be depleted prior to adoptive transfer with resulting losses in cell count. We therefore prefer a process that results in a cell product of high purity, as is achieved by stimulation with Ko-Op. That no depletion step would be necessary after stimulation with Ko-Op is demonstrated by the following calculation example using the percentage data from the 12 donors of **Figure 1C**. Assuming that 3.0x10^6^ stimulated MNC per kilogram body weight are to be transplanted, we would administer an average of 2.77x10^6^ γδ T cells per kilogram body weight (equivalent to 92.19% γδ T cells of MNC) on day 10/11 of cultivation, which in our experience would be an appropriate cell number (6, 16). The number of αβ T cells thereby administered would be 1.14x10^5^ αβ T cells per kilogram body weight (equivalent to 3.81% αβ T cells of MNC). On day 14/15, we would even administer only 5.97x10^4^ αβ T cells per kilogram body weight (equivalent to 1.99% αβ T cells of MNC). We know from *in vivo* studies with NK cells that αβ T cell numbers lower than 5x10^5^ per kilogram body weight are tolerable (31, 32). In only one of the 12 donors was the percentage of αβ T cells too high on day 10 of stimulation, but by day 14 the percentage would have been within the acceptable range. Therefore, MNC stimulated with Ko-Op should be usable without the need for an additional purification step.

Beside bisphosphonate-based protocols, K562 feeder cell-based methods with or without zoledronate, have been developed to expand γδ T cells for cellular therapy in recent years (33, 34). Since this provides sustained stimulation over several weeks, expansion rates are very high and in a direct comparison also higher than with a bisphosphonate-based protocol (33). However, the use of feeder cells increases the complexity of the process and may reduce the chance of regulatory approval. In addition, the K562-based protocol is under patent protection. In conclusion, our newly developed protocol can be more rapidly implemented in the clinic and is more easily applicable for broader use in academia or industry.

### The optimal donor and time schedule for *ex vivo* expansion of γδ T cells with Ko-Op

Adoptive transfer of haploidentical γδ T cells is feasible without limiting side effects (6, 8). Therefore, if several donors are available the question of selecting the most suitable donor arises. Despite our promising results presented here, we see differences between the various donors in terms of yield and anti-tumor activity when we stimulate their MNC *in vitro* using the new cultivation procedure Ko-Op. We found a weak but significant inverse relationship between age and degranulation and a positive correlation between percentage of γδ T cells of unstimulated MNC and proliferation rate of γδ T cells. This is consistent with the finding that γδ T cells from older people do not respond well to IPP (35). Our results also fit the finding of Sutton et al. that the initial γδ T cell number correlates with the final γδ T cell number (29). As consequence, we would choose the youngest adult donor with the highest percentage of γδ T cells of unstimulated MNC to achieve the best possible yield and anti-tumor activity of cells.

The question remains as to the optimal culture duration and timing for adoptive cell transfer. Yield and purity as well as anti-tumor activity should be as high as possible. In view of all the findings, we consider the resulting cell product on day 10 to be the most suitable of the tested days for *in vivo* application and thus also for our further *in vitro* investigations. This is in line with others who also see an optimal outcome at day 10 of *ex vivo* stimulation also in terms of a lower proportion of terminally differentiated cells. However, their stimulation protocol differs from ours as they used K562 artificial antigen presenting cells in combination with zoledronate to expand γδ T cells (36).

### Modulating Ko-Op by pulsing zoledronate and addition of IL-15

The benefits of pulsing zoledronate or addition of IL-15 for expansion and stimulation of γδ T cells have been demonstrated several times (8, 19–21). The rationale for washing out zoledronate after 4h is to reduce cellular toxicity by the aminobisphosphonate (19, 37). But in contrast to others, we saw no benefit regarding the proliferation and the purity when adjusting the Ko-Op protocol by pulsing of zoledronate. The purity was even reduced. However, we already achieved an excellent purity with Ko-Op standard, which could explain the missing benefit of pulsing zoledronate. Additionally, the toxicity of remaining zoledronate does obviously not prevail the possible benefit of not washing out. Indeed, by pulsing, both the culture medium of the first four hours with possibly already released cytokines contributing to the stimulation and the stimulant zoledronic acid itself are lost. However, an increase of intrinsic cytotoxicity of γδ T cells can be achieved by pulsing zoledronate. Furthermore, pulsing zoledronate leads to a higher proportion of TCM, which is associated with a more effective tumor control *in vivo*, at least in αβ T cells studies (38, 39).

The impact of combining IL-2 with IL-15 for γδ T cell stimulation on the proliferation rate is controversial. Van Acker et al. described enhancing effects on proliferation (20). Xu et al. also observed a higher proliferation rate, but they additionally stimulated with vitamin C (8). Like us, Aehnlich et al. and Burnham et al. did not find a benefit (21, 40). Regarding the intrinsic and antibody-dependent cell-mediated cytotoxicity, we unexpectedly only saw benefits of combining IL-2 and IL-15 when zoledronate was pulsed. With respect to the current literature, we would have expected also an enhancing effect if IL-2 and IL-15 is combined with our Ko-Op standard protocol. However, for the intrinsic and the obinutuzumab-mediated cytotoxicity, we could observe higher lytic units when IL-2 was combined with IL-15. Here, because of the high variability of the results of single experiments, a significance level was not reached. IL-15 has already been safely administered in clinical trials, facilitating GMP-compliant use (41).

We also tested the use of IL-15 instead of IL-2 for expansion and found significantly reduced proliferation, purity, cytoplasmic perforin and cytotoxicity. Interestingly, we observed an increased intrinsic cytotoxicity when pulsing zoledronate was combined with IL-15 instead of IL-2. However, because of the small recovery, we do not consider it practical for clinical use to stimulate with IL-15 only.

In summary, pulsing zoledronate and additional administration of IL-15 modifies the anti-tumor activity and the differentiation of stimulated γδ T cells. While enhanced cytotoxicity and a higher percentage of TCM can be considered as improvements, the question arises whether the lower purity by pulsing zoledronate is acceptable for clinical-scale application. This might possibly require an additional depletion step, with corresponding cell loss, before their adoptive transfer. Still, the cultivation procedure for the γδ T cell product can be adjusted to the actual requirements of the clinical application.

## Conclusions and outlook

With this study, we have achieved our goal of establishing an expansion protocol for γδ T cells that is easy to scale up and convert to a GMP-compliant process. We also demonstrated that it is superior to our previous standard procedure with respect to almost all target parameters considered. Furthermore, we examined our results for correlations with donor characteristics in order to draw conclusions about a suitable donor for *ex vivo* expansion followed by haploidentical transplantation. Finally, we demonstrated that the expansion protocol Ko-Op can be further modified by pulsing zoledronate and combining IL-15 and IL-2 as co-stimulators. Further tests with frozen and thawed stimulated cells and *in vivo* studies remain necessary to verify whether the results obtained in this study are reflected by the anti-tumor activity *in vivo*.

## Data availability statement

The raw data supporting the conclusions of this article will be made available by the authors, without undue reservation.

## Ethics statement

The studies involving human participants were reviewed and approved by the Institutional Review Board of the Paracelsus Medical University Nuremberg. Written informed consent to participate in this study was provided by the participants. All donors signed an agreement according to General Data Protection Regulation.

## Author contributions

AB designed the experiments, performed research, analyzed and interpreted data and wrote the paper. HG performed research, interpreted data and edited the paper. EH performed research. SK edited the paper. TH contributed to conception and design, interpreted data and edited the paper. MW contributed to conception and design, interpreted data and edited the paper. These authors contributed equally to this work and share last authorship: TH and MW.

## Supplementary material

Supplementary Figure 1 Percentage of NK cells, vitality of MNC and percentage of perforin^+^ and IFNγ^+^ γδ T cells after *ex vivo* stimulation. MNC of healthy donors were isolated and stimulated with Zol/IL-2 according to the protocols R10F (blue hatched bars) and Ko-Op (orange bars) up to 17 days. **(A)** Representative FACS analysis of MNC cultured according to the protocols R10F or Ko-Op for ten days. NK cells were defined by using anti-CD56-PC5 and anti-CD3-ECD. **(B)** Percentage of NK cells at different days of cultivation measured by flow cytometry. **(C)** Vitality of MNC at different days of cultivation determined by trypan blue exclusion. **(D)** Cytoplasmic perforin and IFNγ in γδ T cells were stained at different days of cultivation and measured by flow cytometry. The data are presented as mean ± SD of 12 **(B, C)** or 9 **(D)** independent experiments. *p<0.05 and **p<0.01 comparing the two different stimulation protocols.
