# Supplementary figures and images for: An optimized cultivation method for future in vivo application of γδ T cells
Source: Front Immunol. 2023 Jul 19;14:1185564. doi: 10.3389/fimmu.2023.1185564 (PMC10394837; doi:10.3389/fimmu.2023.1185564)

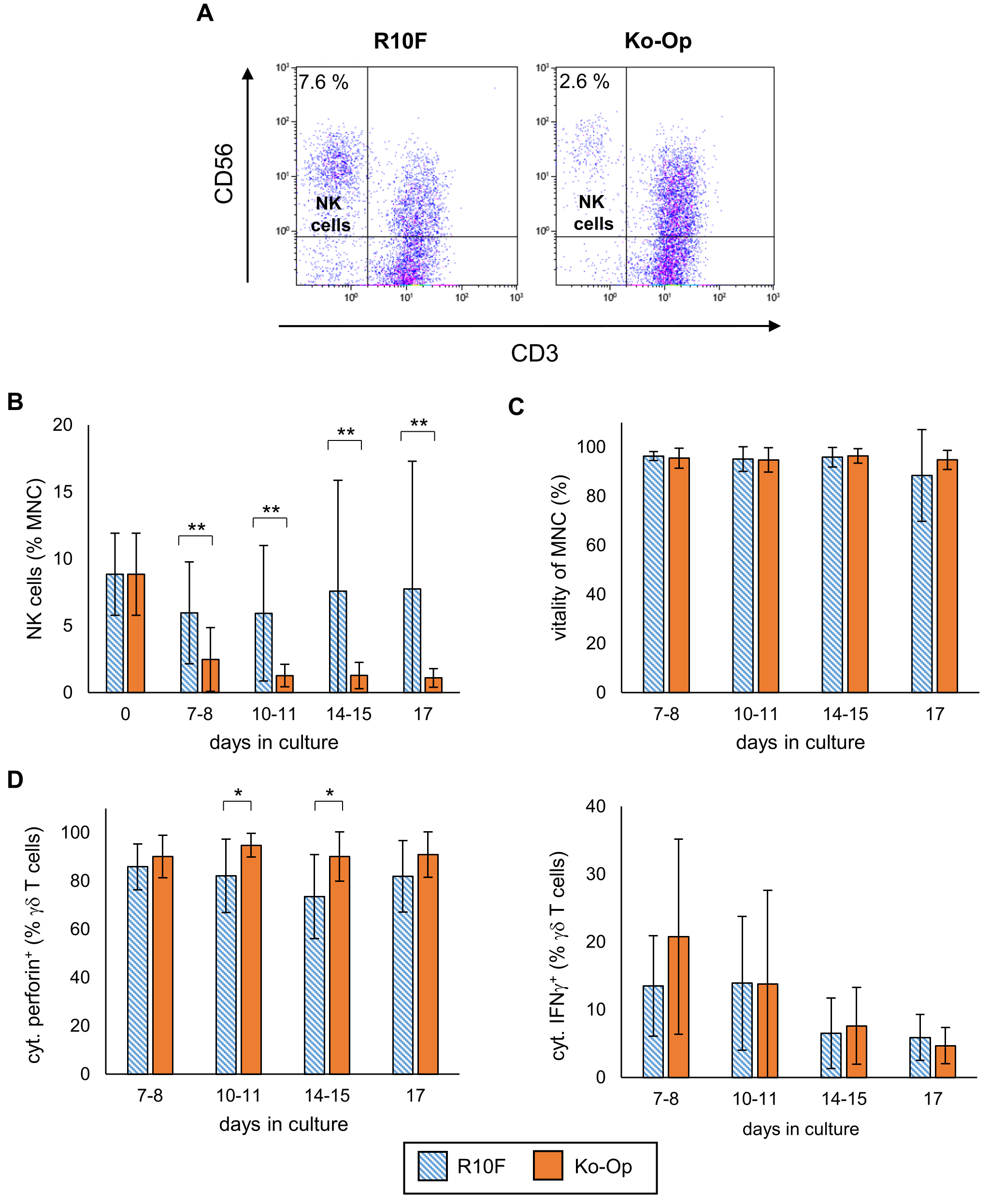

Supplement: Supplementary Figure 1 — Percentage of NK cells, vitality of MNC and percentage of perforin+ and IFNγ+ γδ T cells after ex vivo stimulation. MNC of healthy donors were isolated and stimulated with Zol/IL-2 according to the protocols R10F (blue hatched bars) and Ko-Op (orange bars) up to 17 days. (A) Representative FACS analysis of MNC cultured according to the protocols R10F or Ko-Op for ten days. NK cells were defined by using anti-CD56-PC5 and anti-CD3-ECD. (B) Percentage of NK cells at different days of cultivation measured by flow cytometry. (C) Vitality of MNC at different days of cultivation determined by trypan blue exclusion. (D) Cytoplasmic perforin and IFNγ in γδ T cells were stained at different days of cultivation and measured by flow cytometry. The data are presented as mean ± SD of 12 (B, C) or 9 (D) independent experiments. *p<0.05 and **p<0.01 comparing the two different stimulation protocols. [file Image_1.jpeg]
